# A behavioral approach to annotating sleep in infants: Building on the classic framework

**DOI:** 10.14814/phy2.15178

**Published:** 2022-02-12

**Authors:** Renée A. Otte, Xi Long, Joyce Westerink

**Affiliations:** ^1^ Philips Research Department of Personal and Preventive Care Eindhoven The Netherlands; ^2^ Department of Electrical Engineering Eindhoven University of Technology Eindhoven The Netherlands; ^3^ Philips Research Department of Digital Engagement, Behavior and Cognition Eindhoven The Netherlands; ^4^ Department of Industrial Engineering and Innovation Sciences Eindhoven University of Technology Eindhoven The Netherlands

**Keywords:** behavioral observation, behavioral states, infants, sleep annotation, unobtrusive sleep monitoring

## Abstract

In infants, monitoring and assessment of sleep can offer valuable insights into sleep problems and neuro‐cognitive development. The gold standard for sleep measurements is polysomnography (PSG), but this is rather obtrusive, and unpractical in non‐laboratory situations. Behavioral observations constitute a non‐obtrusive, infant‐friendly alternative. In the current methodological paper, we describe and validate a behavior‐based framework for annotating infant sleep states. For development of the framework, we used existing sleep data from an in‐home study with an unobtrusive test setup. Participants were 20 infants with a mean age of 180 days. Framework development was based on Prechtl's method. We added rules and guidelines based on discussions and consent among annotators. Key to using our framework is combining data from several modalities, for example, closely observing the frequency, type, and quality of movements, breaths, and sounds an infant makes, while taking the context into account. For a first validation of the framework, we set up a small study with 14 infants (mean age 171 days), in which they took their day‐time nap in a laboratory setting. They were continuously monitored by means of PSG, as well as by the test setup from the in‐home study. Recordings were annotated based both on PSG and our framework, and then compared. Data showed that for scoring wake vs. active sleep vs. quiet sleep the framework yields results comparable to PSG with a Cohen's Kappa agreement of ≥0.74. Future work with a larger cohort is necessary for further validating this framework, and with clinical populations for determining whether it can be generalized to these populations as well.

## Graphical abstract

We developed a new framework for annotating behavioral states in infants. It is based on existing frameworks, and extends these. We ran a small lab study as a first validation of the framework.

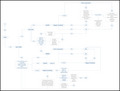

## 1 INTRODUCTION

During their infant years, children spend more time asleep than awake. This makes sleep the dominant activity of their rapidly developing brains (El‐Sheikh & Sadeh, 2015; McKenna et al., 1993). When asleep, for instance, memory consolidation, memory reorganization, and lexical development take place, and semantic knowledge is created (Friedrich et al., 2015, 2017; Gómez et al., 2006). Evidence has suggested that differences in sleep efficiency may be associated with different trajectories of cognitive development (Pisch et al., 2018). In addition, bedtimes and total sleep duration have been linked to differences in social‐emotional development (Mindell et al., 2017). Issues with sleep in infants, therefore, could significantly impact infant development.

Sleep monitoring and the assessment of sleep can offer valuable insights into (potential) sleep problems, and neuro‐cognitive and socio‐emotional development in infants (Acebo et al., 1999; Anders, 1978; Gertner et al., 2002; Sadeh, 1994). As in adults, the gold standard for sleep measurement in infants is polysomnography (PSG; Berry et al., 2015). Because of the many electrodes needed for this method, however, it is rather obtrusive, and impractical in non‐lab and home situations. Also, it has been found to change a subject's normal sleep (Horne & Biggs, 2013). An alternative to PSG is actigraphy, which has been reliably used in normal infant populations for detecting sleep and wakefulness (Sadeh, 2011; Sadeh et al., 1991). Although actigraphy can easily be used outside the lab, it needs to be attached to a wrist or ankle, making it obtrusive still. A non‐obtrusive alternative is to ask parents to fill out sleep diaries for their infants, but this method is subjective, and inaccurate for recording night waking (Galland et al., 2012). Accuracy of sleep diaries can be improved by combining them with actigraphy (Horne & Biggs, 2013), but then we have come full circle to obtrusiveness again.

The norm before the introduction of PSG and actigraphy was behavioral observations of the sleeping child, which researchers have shown to be reliable in trained annotators (Grigg‐Damberger et al., 2007). Although often time‐consuming, behavioral observations constitute a non‐obtrusive, infant‐friendly method for measuring their sleep. Several scales and methods for annotating sleep in infants based on behavior have been developed, for example those by Prechtl (1974) and by (Anders & Chalemian, 1974; for a review of alternative techniques to study infant sleep see Grigg‐Damberger et al., 2007). Prechtl (1974) classified five states based on observations of the eyes (open vs. closed), respiration (regular vs. irregular), movements (yes/no), and vocalization (yes/no). Anders and Chalemian (1974) defined four states of wakefulness, and two sleep states, also based on observations of movements, vocalizations, respiration, and the eyes of the infant. Despite their advantages for infant populations, these methods present two drawbacks that, as a consequence, decrease their reliability. First, some types of behavior that provide the annotator with information essential for correctly discriminating between sleep states are difficult to assess. For instance, visually observing the respiration of an infant without any instrumentation has been shown to be a complex and unreliable process (Anders et al., 1971). Second, precise descriptions of what those types of behavior look like are often unavailable. As an example, Prechtl describes that both in states 2 and 4 infants can make head, arm or leg movements. However, he does not explain how these movements differ between the two states, so how they can help you discriminate between them. Therefore, we developed a method for annotating sleep in infants that is based not only on video and audio recordings, but also on a non‐obtrusive sensor for recording movement and respiratory data. Importantly, our method includes detailed descriptions of the type and quality of movements infants make when sleeping.

The goal of this paper was two‐fold. Our main purpose was to introduce the behavioral framework we have developed for annotating sleep states in infants below one year of age. This is the topic of Section 2: Framework development. Second, in Section 3, Framework validation, we present results from a small study in which we did a preliminary validation of our framework against PSG.

## 2 FRAMEWORK DEVELOPMENT

### 2.1 Methods

For developing our behavioral sleep annotation (BSA) framework, we used data that had been collected during an in‐home observational study that focused on sleep and sleeping problems in infants below one year. Below, we describe this study (further referred to as “home study”), and how the BSA framework was developed from it. In the Results Section (2.2) we present the framework itself.

#### 2.1.1 Collection of infant sleep data

We collected nocturnal sleep data from 20 infants (M age = 180 days, range 88–250 days, 10 boys), in their own home, through a setup with non‐obtrusive instruments. Parents had been recruited through flyers, word‐of‐mouth, and the researchers’ networks. The study had been approved by both the Psychological Ethical Test Committee (PETC) of Tilburg University and the Internal Committee on Biomedical Experiments (ICBE) of Philips Research. Informed consent had been obtained from all parents in accordance with the declaration of Helsinki.

The setup consisted of (1) an infrared video camera with a wide‐angle lens overlooking the whole crib, (2) an audio recorder, and (3) a custom‐made piezo pressure sensor underneath the mattress that measured body movements and ballistocardiography (BCG). Respiration can be obtained from BCG with relatively reliable accuracy (Werth et al., 2017). For example, researchers have reported an average error of 2.53% compared to a reference breathing sensor in healthy infants (Lee et al., 2015). Signals from all devices were time‐locked to each other, and stored on a silent PC that was placed underneath the crib. Parents turned the setup on before putting their baby in bed at night, and turned it off after taking their baby out of bed the next morning. For each infant we recorded 14 nights of sleep, retaining about 1600 h of sleep after cleaning up the data, that is, after removing parts of the data in which sensors malfunctioned or the infant was just out of scope of the camera.

#### 2.1.2 Development of the framework

For annotation of the data from the home study, we started by using the method developed by Prechtl (1974). It uses “states” to refer to a descriptive classification of infant behavior that corresponds to distinct modes of infant brain activity. The states are described as vectors consisting of particular properties, which together form a finite and discrete vector space. The four relevant properties for this vector space are “eyes open,” “respiration regular,” “gross movements,” and “vocalization.” By combining them, it is possible to distinguish between five behavioral states, see Table 1.

**TABLE 1:** Vectors of behavioral states as defined by Prechtl (1974)

	Eyes open	Respiration regular	Gross movements	Vocalisation
State 1: Quiet sleep (QS)	−	+	−	−
State 2: Active sleep (AS)	−	−	−	−
State 3: Quiet awake (QA)	+	+	−	−
State 4: Active awake (AA)	+	−	+	−
State 5: Vocalization (V)	0	−	+	+

Note

A minus refers to the absence of a property for a state, a plus refers to the presence of a property for a state, and a zero indicates that a property may either be absent or present for a state.

We based our approach on Prechtl's work, because it provides a simple delineation between the different infant behavioral states. In addition, as we were mainly interested in sleep states, Prechtl's work suited our purposes better than, for instance, the method by Anders and Chalemian (1974), as they also classified four states of wakefulness.

While scoring the sleep data collected in the home study, we noticed that infants in our population behaved differently from Prechtl's original descriptions. For instance, we observed gross movements (i.e., limbs moving) during AS, and occasionally even during QS (e.g., turning over). Also, discrimination between states was sometimes difficult, especially during transitions from AA or QA to AS (i.e., on falling asleep), or from AS to QS or vice versa. We therefore generated extra rules and guidelines for ambiguous sections within the sleep recordings. Over time, these were discussed among the annotators, based on examples from the data, and fine‐tuned to be as specific as possible. This process resulted in an elaborate set of rules, observations, characteristics, and guidelines to be used in addition to Prechtl's vectors, which we formalized into a framework so that others may use it in their work, too. In the next section we present the framework.

### 2.2 Results

**FIGURE 1:**
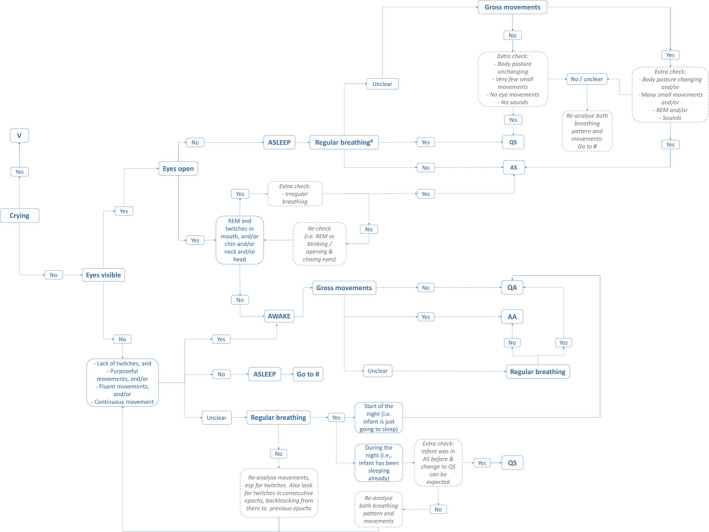
Graphical representation of the behavioral sleep annotation (BSA) framework

The data we collected in the in‐home study were used for the development of the behavioral sleep annotation (BSA) framework, of which a graphical representation can be found in Figure 1 (a larger version is included in the Appendix 1). In the next sections, we will illustrate how to use this framework. We will start with describing some of its basic design choices, continue with a description of how to identify and discriminate between different states, and conclude with a description of how to take the context into account.

#### 2.2.1 BSA framework basics

In general, information from several modalities is combined to decide on which state the infant was in: respiration, movement, audition. The epoch length for annotation is 30 s, which is in line with the AASM Pediatric Task Force (Grigg‐Damberger et al., 2007). When—during transitions—two states occur within one epoch, the state comprising the greatest portion of the epoch is assigned, also in accordance with the AASM’s Pediatric Task Force (Grigg‐Damberger et al., 2007).

#### 2.2.2 Discriminating between wake and sleep

When annotating, the eyes are among the most important features for distinguishing between wake and sleep: when an infant's eyes are open, they are awake; when their eyes are closed, they are asleep (Anders & Chalemian, 1974; Anders et al., 1971; Prechtl, 1974). In practice, however, there are a number of difficulties with this: (1) some infants go through a period of opening and closing their eyes before they fall asleep, for example, for up to 10 min, making it hard to decide on which epoch to annotate as the first sleep epoch; (2) some infants immediately go to slow or rapid eye movement sleep on falling asleep after a period of opening and closing their eyes, again making it difficult to decide on the first sleep epoch; (3) some infants sleep with their eyes (partially) opened; (4) sometimes an infant's eyes are invisible, for example, they are covered with an arm or blanket. In ambivalent cases such as these, that is, when one cannot rely on the simple principle of ‘eyes open = awake’ and “eyes closed = asleep,” we used additional information about movements to determine wake or sleep. That is, first we looked for twitches in hands, arms or the face, which are characteristics of sleep and not of wakefulness (see e.g. Kahn et al., 1996). Second, we decided on goal‐directedness or “purposefulness,” for example, an infant voluntarily reaching for toy, versus them making an involuntary, random movement. The first type of movements is associated with being awake, and the second with being asleep (see also Schwichtenberg et al., 2018). Third, we studied fluency, where during wakefulness infant movements are more fluent, and during sleep they are more jerky. Finally, we took frequency into account, with sleep being associated with more and longer intervals between consecutive movements than wakefulness. See Table 2 for a summary of the observations described above.

**TABLE 2:** Summary of movement observations that can be used to discriminate between wake and sleep

#	Type of movement	Wake	Sleep
1	Twitches	No	Yes
2	Goal‐directed/with purpose	Yes (most of the time)	No (more at random)
3	Fluency	Yes (fluent movements)	No (jerky movements)
4	Frequency	More movements, and more continuous movements	More and longer intervals between movements

#### 2.2.3 Discriminating between quiet sleep and active sleep

**FIGURE 2:**
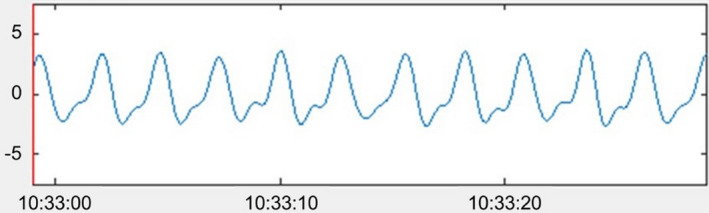
Screenshot from a custom Matlab tool with an example of a signal showing regular breathing

**FIGURE 3:**
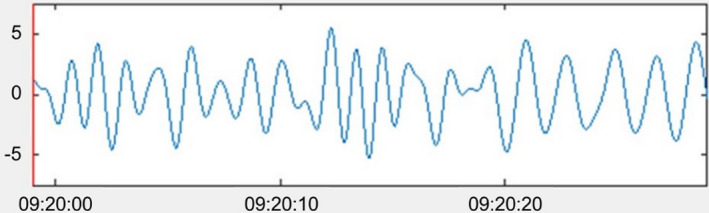
Screenshot from a custom Matlab tool with an example of a signal showing irregular breathing

For making the distinction between quiet sleep (QS) and active sleep (AS), Prechtl described respiration to be the main discriminating factor (see also Table 1): regular respiration corresponds to QS and irregular respiration corresponds to AS. Regularity of respiration can be determined by visual inspection of the respiration signal: in regular respiration the frequency and amplitude of the breathing signal remain similar throughout an epoch, while in irregular respiration these change frequently, and apnoeic spells may be seen (Prechtl, 1974). To be more precise, when visually inspecting an epoch and extrapolating the respiratory rate for a minute, if the rate in the longest and shortest cycles varies less than 20 cycles/min, respiration is regular. If it varies more than 20 cycles/min, respiration is irregular (Anders et al., 1971). See Figures 2 and 3 for examples of both regular and irregular respiration, respectively.

Respiration, however, is not always sufficient and certainly not the only factor useful for making the distinction between QS and AS. If from the respiration signal it is unclear in which sleep state an infant is, the second most important factor to consider is their movement: its type and quality. That is, during QS there is very little movement (see also e.g., Anders & Chalemian, 1974; Davis et al., 2004; Kahn et al., 1996; Prechtl, 1974; Sheldon, 2006), infants mainly remain in the same body posture for the duration of the state, and only very few twitches or other small movements can be observed. Occasionally infants may turn over (e.g., from back to belly), and this is accompanied by irregular respiration. However, respiration then returns to regular within a few epochs (i.e., 1–5, see also Prechtl, 1974).

During AS, on the other hand, there is a lot of movement, from gross movement to small twitches and startles to eye movements (again, see e.g., Anders & Chalemian, 1974; Davis et al., 2004; Kahn et al., 1996; Prechtl, 1974; Sheldon, 2006). Movements often occur in a cyclic pattern, that is, they are separated by intervals of a few minutes that generally last for approximately the same duration (Prechtl, 1974). Periods of prolonged movement, for example, 5–10 min, can also be observed in which there are fewer or only very short intervals between the movements. A summary of the characteristics to discriminate between QS and AS can be found in Table 3.

**TABLE 3:** Characteristics of quiet and active sleep that can be used to discriminate between the two states

#	Characteristic	Quiet sleep	Active sleep
1	Respiration	Regular	Irregular
2	Movement		
2a	Body posture	Mainly unchanging	Changing regularly
2b	Gross and small body movements	Seldom	Often (e.g., limb displacement; writhing movements; stretching; twitches in mouth, chin, neck, head, fingers, toes; (one‐sided) smiles; grimaces; frowns; sucking movements)
2c	Eye movement	Seldom	REM (sometimes with (partially) opened eyes); slow eye movement
3	Sounds	None	Occasionally (e.g., whimpers; moans; cries)

A final factor that can help determine whether an infant is in QS or AS pertains to sleep cycles. Infant sleep cycles last about 50 to 60 min, with relatively more active than quiet sleep the younger the infant is (Carskadon & Dement, 2005; Kahn et al., 1996). The longer an infant has been in one sleep state the larger the chance becomes that they will transition to the next state. For example, after a period of about 20 to 30 min of AS the annotator may expect a transition to QS. As QS and AS alternate within a cycle this can help in predicting when a transition between QS and AS will take place, and in checking whether any transitions may have been missed.

#### 2.2.4 Discriminating between quiet awake and active awake

The most important factor to determine whether an infant is in the quiet awake (QA) versus the active awake (AA) state is movement. QA, on the one hand, is associated with very little movement, except for eye movement, and unchanging body posture. AA, on the other hand, is associated with movements of the arms, legs, and head (Prechtl, 1974). In AA eye movements are mainly observed when the infant pauses movement with the rest of his body. In addition to movement inspection of the respiration signal can help distinguish between the two states, with respiration being regular when an infant is in the QA state, and irregular when they are in AA. Note that as of all states infants spent least time in state QA (Prechtl, 1974) there may only be a few QA epochs in every recording.

#### 2.2.5 Identifying vocalisations

As Prechtl (1974) described, the main characteristic of state 5 is crying. For obvious reasons, the audio signal will provide essential information for deciding whether or not an infant is crying. However, since different infant sounds may sound quite similar, the video recordings the audio signal is time‐locked to provide invaluable information for discriminating between crying and non‐crying. For example, we annotated epochs in which it sounded like the infant was crying, when in fact the video recording showed them to be giggling loudly about something—and the other way around. Thus, V should never be annotated based on the audio signal only.

Note that vocalizations can occur during both periods of wakefulness and sleep. Also, note that V is only annotated when an infant is actually crying. When they are laughing, moaning or whimpering the other states are used for annotating the epochs concerned, that is, AA, QA, AS, or QS.

#### 2.2.6 Taking the context into account

A number of rules guide the way in which the context of previous and consecutive annotations are taken into account, see Table 4.

**TABLE 4:** Annotation rules for taking the epoch context into account

Rule #	Description
1	Only accept changes lasting longer than 3 min as a new state
1a	V episodes may be annotated even if they last for less than 3 min
1b	On falling asleep AA, QA, and AS may be annotated even if they last for less than 3 min
2	When a transition between states occurs only start with annotating the new state after the transition is complete
3	QS cannot follow AA or QA, and AA and QA cannot follow QS

We developed these rules because sleep is cyclic, and it is unlikely that infants switch from one state to the next and back from epoch to epoch (Anders et al., 1971; Aserinsky & Kleitman, 1955; Dement & Kleitman, 1957; Prechtl, 1974). Rule 1 is related to this and based on Prechtl’s (1974) paper: only changes lasting longer than 3 min are accepted as a new state. An exception to this rule exists for the V annotation: based on consensus between annotators vocalization episodes are also annotated as such if they last for less than 3 min (Rule 1a). In addition, when falling asleep annotation shorter than 3 min is allowed as a new state (Rule 1b; see also Grigg‐Damberger et al., 2007). This is because infants are often drifting from AA or QA to AS and back within 3 min. As the vector states described by Prechtl do not cover a drowsy state, in these specific cases of falling asleep states shorter than 3 min are allowed.

Second, in a transition between two states the current state is annotated until the transition to the new state is complete (Rule 2), and not annotated as “indeterminate sleep.” There is prior work suggesting transitions between states play a role in sleep development (e.g., Thoman, 1990; Weisman et al., 2011). However, we chose to follow the Pediatric Task Force of the AASM (Grigg‐Damberger et al., 2007). who recommended not routinely annotating indeterminate sleep. They base this recommendation on studies showing poor inter‐rater reliability for indeterminate sleep. As an illustration of Rule 2, we regularly observed a transition between two states that lasted for several epochs, for instance when the respiration of an infant in AS gradually became more regular, and the infant started moving less. As described by Anders at al. (1971) and Curzi‐Dascalova et al. (1981) we, too, noticed that this mostly happens when moving from AS to QS; the transition from QA to AS is more abrupt.

Finally, QS cannot follow AA or QA, and vice versa (Rule 3), since healthy infants over 2–3 months generally do not transition from wakefulness to QS or from QS to wake directly (see e.g. Barbeau & Weiss, 2017; Grigg‐Damberger et al., 2007; McCormick & Westbrook, 2013). Therefore, if such a transition does appear to happen we assume this to be annotator error and we advise re‐annotating the part of the sleep recording concerned.

#### 2.2.7 Additional guidelines

When reading the previous sections it quickly becomes clear that annotating behavioral states in infants can at times be quite challenging. Therefore, we have formulated additional guidelines. The first is about movement (Guideline 1). Infant movement during sleep represents noise in the respiration signal. On the one hand, this can make it more difficult to judge whether respiration is regular or irregular. On the other hand, the noise in itself yields information about the state an infants is in. That is, when there is a lot of movement noise in the respiration signal it is unlikely the infant is in one of the quiet states (QA, QS), but all the more likely they are in one of the active states (AA, AS).

The second is about play back speed of sleep recordings (Guideline 2). Inspecting infant sleep recordings at higher speeds can help assess what type of movements an infant is making, and thus help distinguish between states (Anders & Keener, 1985; Anders & Sostek, 1976). For instance, at higher speeds it becomes easier to spot small movements or twitches. Also, jerky, random movements indicative of AS can be discriminated more easily from fluent goal‐directed movements pointing to wakefulness. We advise using 8x normal speed, since this has the double advantage of considerably speeding up the annotation work, while still being able to observe what is happening in the recording. At higher speeds it becomes increasingly difficult to see what is happening, and the chance of missing events and making annotation mistakes increases. Note that in case of uncertainty, for example, when a transition is taking place, we advise slowing the recording down to normal speed.

Finally, every baby is different, including the way they move, and thus the same state may look quite different in different babies. For example, in our study, one infant made small twitching movements in all sleep states, which made it difficult to assess whether or not she had transitioned from AS to QS. Here, we relied heavily on information about body posture and gross movement to determine her sleep state. Another infant almost did not move in her light sleep, so we needed to base ourselves almost solely on the respiration signal. Thus, a learning curve may be expected for each infant that is new to an annotator (Guideline 3). However, this does not imply that if things become difficult anything goes. What it does imply is that for some infants the annotator may need to rely more on one modality, and for other infants on another. A summary of the guidelines formulated here can be found in Table 5.

**TABLE 5:** Additional guidelines that may help the annotator discriminate between states

Guideline #	Description
1	Movement noise in the respiration signal represents information about the infant's sleep state
2	Inspecting the video recordings at 8x normal speed helps speed up annotation and facilitates observation and interpretation of motion
3	Each infant is different so a learning curve in annotating may be expected for each new infant

#### 2.2.8 Tooling for annotation

When annotating behavioral states in infants we suggest using a tool in which signals from different modalities are synchronized, and can be inspected epoch by epoch. Minimal requirements are time‐locked (Anders et al., 1971) video, audio, and respiratory data. We used a custom‐made Matlab tool in which the video recordings were shown per epoch and could be played at different speeds. Audio data, and movement and respiration data from the piezo sensor were represented in a waveform. When playing the video, a marker showed the corresponding position in the movement, respiration, and audio signal, respectively. In addition, the tool showed context information such as the subject's ID, the date of the sleep recording, the current position in the recording, and the epoch number. Note that the tool was just that, a tool. That is, it was not programmed to do annotations automatically; those were done manually by the annotators.

**FIGURE 4:**
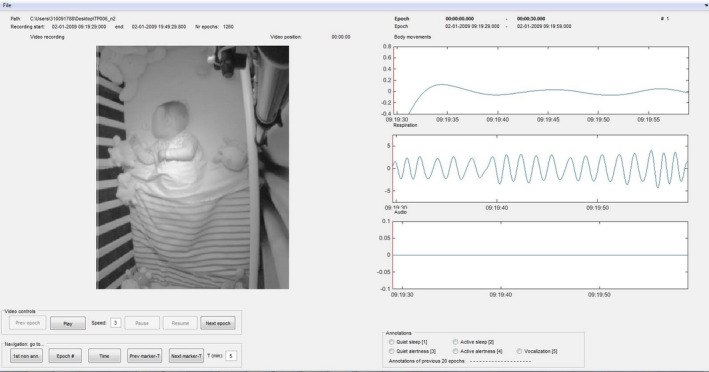
Screenshot of a custom Matlab tool for annotating sleep data. Note that for privacy reasons the face of the infant has been blurred

To more easily take the context of an epoch into account, and to find transitions between states, in addition to the epoch‐by‐epoch video functionality in the tool we inspected the video for the complete sleep recording in a media player. A screenshot of the tool we used can be found in Figure 4.

## 3 FRAMEWORK VALIDATION

To validate our framework we tested it with 13 infants in a pilot study in a laboratory setting. We recorded a morning or afternoon nap by means of both PSG and the non‐obtrusive test setup from the home study, with time‐locked audio, video, and movement/respiration signals.

### 3.1 Methods

#### 3.1.1 Participants

Thirteen healthy infants (age 2–10 months, 9 boys) took part in the lab study in the Babylab of Tilburg University, The Netherlands. The study was approved by both the Psychological Ethical Test Committee (PETC) of Tilburg University and the Internal Committee on Biomedical Experiments (ICBE) of Philips Research. Informed consent was obtained from all parents in accordance with the declaration of Helsinki.

Parents of the infants had been recruited: (1) through flyers aimed at young parents living in or close to Tilburg; (2) through word‐of‐mouth; (3) through the website of the Tilburg University Babylab, and (4) through meetings for expecting couples in the maternity ward of St. Elisabeth Hospital in Tilburg, The Netherlands.

Data from two subjects were excluded from further analyses due to missing audio recordings—and therefore missing observational annotations. Data from a third infant were excluded because the net with electroencephalography (EEG) sensors had been displaced, and therefore PSG could not be annotated reliably. The remaining 10 infants (6 boys) were aged 173 days on average (range 66–279 days). They were healthy and had been born at term (gestational age ≥37 weeks).

#### 3.1.2 Procedure

Data recording took place in a dimly lit and sound‐attenuated room of the Tilburg University Babylab. Infants and their parents came in around the time the infant normally took their morning, mid‐day or afternoon nap. They slept in a standard‐sized crib (60 × 120 cm) provided by the research team, in their own pajama or sleeping bag, and with their usual sleep toys. Parents followed the usual before‐bed rituals as much as possible, and stayed in the room next door during their infant's nap, with the researchers, until they woke up.

During their nap infants were continuously monitored by means of PSG and behavioral observation. PSG was recorded according to the 2nd version of the guidelines set by the American Academy of Sleep Medicine (AASM; Berry et al., 2015; Grigg‐Damberger et al., 2007). Bipolar EEG was recorded from F3, F4, C3, C4, O1, O2 with electrode positions set according to the international 10–20 system, and re‐referenced to the contralateral mastoid. In addition, eye movements (EOG), muscle tone (EMG), electrocardiogram (ECG), and respiratory rhythm from thorax and abdomen (respiratory inductance plethysmography; RIP bands) were recorded. Also, to collect information for the BSA annotation the same unobtrusive setup as in the home study was used.

#### 3.1.3 Data processing

In total, we collected 9.07 h of data, with an average naptime of 54 (range 32–87 min). Data were inspected in 30s epochs. For each epoch we made both a PSG classification and we annotated it according to the BSA framework.

##### PSG scoring

PSG data were rated by an expert according to the AASM (Berry et al., 2015; Grigg‐Damberger et al., 2007) into the following states: Wake, REM, N1, N2, and N3. As usual, we combined N1, N2, and N3 states (Grigg‐Damberger et al., 2007) into one NREM category. However, we also followed a second combination method used before by Prechtl (1974) and Anders and Chalemian (1974): we merged REM‐epochs together with N1‐ and N2‐epochs into one REM^+^ category, and treated N3 as its opposed category NREM^−^.

##### BSA Scoring

Two annotators independently inspected the video, audio and movement recordings, and the non‐obtrusive respiratory data. The infant behavioral states were annotated according to the BSA framework (state 1 to 5). For each epoch this yielded an AA, AS, QS, QA, or V annotation. To be able to compare these annotations to the PSG data, in accordance with the description by Anders and Chalemian (1974) we merged all epochs annotated as state QA, AA, and V into one “awake” state. This ultimately yielded 3 relevant states: “awake,” “active sleep (AS),” and “quiet sleep (QS).” Note that in this study, V only occurred in infants that were awake; none of the infants vocalized while they were sleeping.

#### 3.1.4 Analyses

For the BSA framework, we first calculated inter‐rater reliability between the two human annotators using Cohen's Kappa coefficient of agreement for each infant, reporting the mean and standard deviation across infants. We then used Cohen's Kappa for comparing between the annotations by the PSG expert and the behavioral annotations by each annotator based on the two different approaches mentioned previously. We thus compared BSA Wake|QS|AS scores to PSG Wake|REM|NREM scores as well as to PSG Wake|REM^+^|NREM^−^ scores. Additionally, we compared the inter‐rater agreements for each of the sleep states. Statistical significance of the comparisons was examined with a Wilcoxon's two‐sample test, where *p* ≥ 0.05 was considered statistically non‐significant (NS). For multiple comparisons, we applied Bonferroni correction.

### 3.2 Results

**FIGURE 5:**
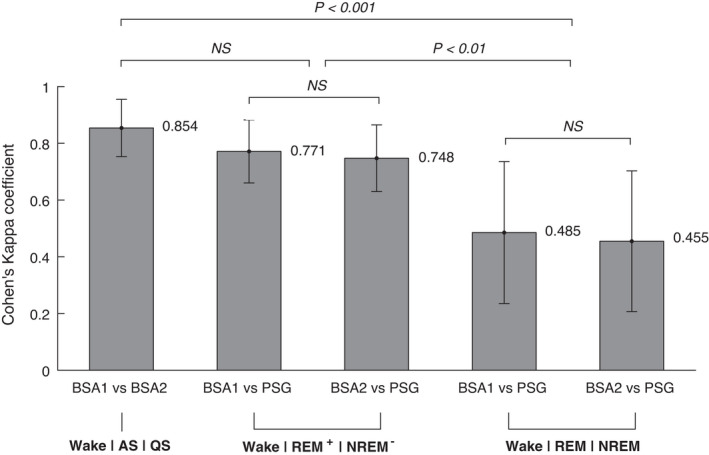
Inter‐rater reliability (Cohen's Kappa coefficient) of the sleep state annotations between two behavioral annotators (BSA1 and BSA2), and between each of them and a PSG expert, for two different merging schemes: Wake|REM^+^|NREM^−^ and Wake|REM|NREM. Here REM^+^ = REM + N1 + N2, NREM^−^ = N3, NREM = N1 + N2 + N3. Mean ± standard deviation results are presented. Significance of difference was examined with the Wilcoxon sign‐rank test, where for multiple comparisons Bonferroni correction was applied

**FIGURE 6:**
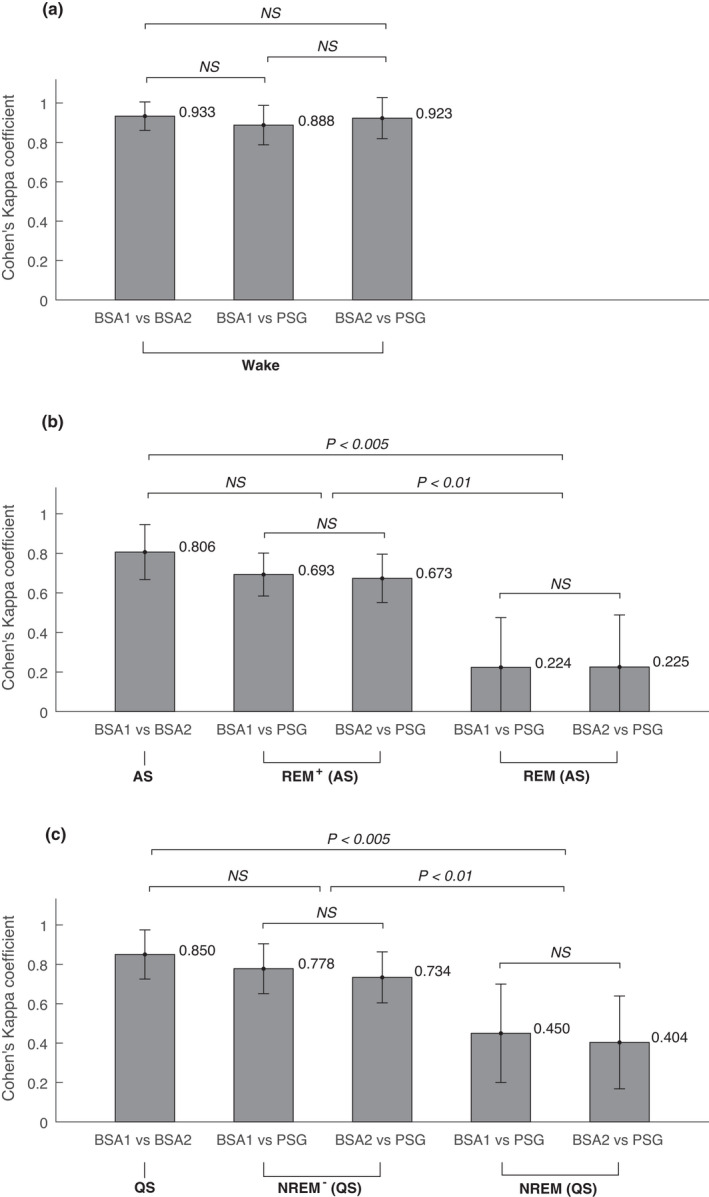
Inter‐rater reliability (Cohen's Kappa coefficient) of annotations per behavioral state between two behavioral annotators (BSA1 and BSA2), and between each of them and a PSG annotator, in (a) Wake, (b) AS (and REM^+^ in scheme A, REM in scheme B), and (c) QS (and NREM^−^ in scheme A, NREM in scheme B). Here REM^+^ = REM + N1 + N2, NREM^−^ = N3, and NREM = N1 + N2 + N3. Mean ± standard deviation results are presented. Significance of difference was examined with the Wilcoxon sign‐rank test, where for multiple comparisons Bonferroni correction was applied

The percentage of sleep states annotated by the PSG expert and the BSA annotators can be found in Tables 6 and 7, respectively. Cohen's Kappa coefficient between the two behavioral annotators (BSA1 and BSA2), and between each of the two BSA annotators and the PSG expert is compared in Figure 5 for both merging approaches described in the Methods section (Wake|REM|NREM and Wake|REM^+^|NREM^−^). Figure 6 depicts the agreement between BSA and PSG annotations for each sleep state.

**TABLE 6:** Percentage of PSG sleep states (mean ± standard deviation)

PSG sleep state	Percentage
Wake	20.8 ± 19.4%
REM	11.4 ± 12.0%
NREM	67.8 ± 20.7%
N1	9.7 ± 13.3%
N2	14.0 ± 6.3%
N3	44.1 ± 19.4%

**TABLE 7:** Percentage of BSA sleep states as measured by annotators BSA1 and BSA2 (mean ± standard deviation)

BSA sleep state	Percentage	
	BSA1	BSA2
V vocalization	5.9 ± 8.2%	9.4 ± 8.8%
AA active awake	17.8 ± 14.6%	12.5 ± 13.3%
QA quiet awake	0.4 ± 0.8%	0.4 ± 0.7%
AS active sleep	32.9 ± 16.6%	39.5 ± 16.9%
QS quiet sleep	43.0 ± 19.7%	38.2 ± 18.7%

As can be seen in Figure 5, the two BSA annotators achieved high inter‐rater reliability in annotating Wake, AS and QS, with a Kappa of 0.854. This was slightly higher than the agreement between the BSA annotators and the PSG expert for Wake|REM^+^|NREM^−^, *p* = 0.031, and significantly higher than the agreement between the BSA annotators and the PSG expert for Wake|REM|NREM (*p* < 0.001). Agreement between the BSA annotators and the PSG expert was also significantly higher for Wake|REM^+^|NREM^−^ than for Wake|REM|NREM (*p* < 0.01).

Figure 6 shows that the inter‐rater reliability between the two behavioral annotators was consistently high for all states (Wake: 0.933 ± 0.072, AS: 0.806 ± 0.139, and QS: 0.850 ± 0.125). For completeness sake we also looked at the inter‐rater reliability for V and AA, each as compared to all other states, which was also consistently high (0.77 and 0.81, respectively). We could not include QA in these analyses, since there were too few QA epochs (see also Table 7).

For the Wake state no significant difference was found between different (behavioral and PSG) annotators. The agreement for both the REM^+^ state (0.693 ± 0.109/0.673 ± 0.122) and the NREM^−^ state (0.778 ± 0.127/0.734 ± 0.130) was substantial, and higher than that for the REM state (0.224 ± 0.251/0.225 ± 0.263) and the NREM state (0.452 ± 0.250/0.404 ± 0.236).

## 4 DISCUSSION

In this paper, we described a framework for annotating infant sleep states based on behavioral observations from video and audio recordings in combination with unobtrusively collected respiration data. We defined rules, observations, characteristics, and guidelines which can be used when annotating sleep in infants based on unobtrusive measurements. Key to using this framework is combining data from several modalities (i.e., respiration, video, audio), taking the context into account (i.e., using information from previous and subsequent epochs), and closely observing the frequency, type, and quality of movements an infant makes while asleep and awake.

In addition to outlining the framework we reported results from a pilot study in which we compared the framework with PSG, the gold standard for annotating sleep in infant, child and adult populations. For comparisons, we used two schemes: Wake|REM^+^|NREM^−^ and Wake|REM|NREM. We found fair to moderate agreement between PSG and BSA for Wake|REM|NREM, that is, Cohen's Kappa values ≥ 0.45, and substantial agreement between PSG and BSA for Wake|REM^+^|NREM^−^, that is, Cohen's Kappa values of ≥ 0.74.

The results of our pilot study correspond with findings from Anders and Sostek (1976), who found high product‐moment correlations between PSG and behavioral annotations based on time lapse video recordings in 2‐ and 8‐week‐old infants. They are also in line with the findings from Kirjavainen et al. (1996) who reported Cohen's Kappa values of 0.67 and higher between PSG annotations and annotations (REM/NREM/Wake) based on recordings from a static‐charge‐sensitive bed. In addition, they provide additional evidence that a behavior‐based annotation framework in general can be used by trained annotators to reliably classify sleep states in infants (see e.g. Becker & Thoman, 1983; Curzi‐Dascalova et al., 1981; Thoman et al., 1976; Thoman & Tynan, 1979).

An advantage of our framework over existing annotation frameworks, such as that by Anders and Chalemian (1974), is that it gives a more detailed and precise description of the behavioral states, especially with respect to movements, and how to differentiate between them. As compared to PSG, an important advantage of the BSA framework is that it is unobtrusive. This makes it suitable for in‐home monitoring of infants, in their natural environment, and is likely to lessen first night effects that may be observed in laboratory settings (Agnew et al., 1966; Grigg‐Damberger et al., 2007). Infants can also be monitored longer term, yielding valuable longitudinal data on their sleep and sleep development. In addition, as no on‐body sensors are required, the framework is promising for premature infants since it would save stressful handling and does not require gels or stickers that may harm their fragile skin (Davis et al., 2015; Montirosso & Provenzi, 2015). Next, it is cheaper than PSG and no medical personnel are required for using it. As such, it becomes financially interesting to reserve PSG for certain cases and fall back on our framework in other cases. At the same time, our framework provides professionals without expensive PSG equipment the opportunity to still annotate infant sleep.

A disadvantage, on the other hand, is that annotation according to our framework is time‐consuming. By in the future automating (parts of) the annotation, as is customary in PSG scoring, annotation time could be reduced considerably. A tool such as the one described in Section 2.2.8 could be used for this. Another disadvantage is that while neither medical equipment nor staff is required for the recordings and the annotations, reliable recording equipment and expertise to use it is still necessary for collecting the audio, video, and respiratory signals in such a way that they can be synchronized perfectly. Furthermore, the BSA framework does not allow for scoring drowsy states. This point could be solved by moving away from the vectors described by Prechtl (1974), and adding a drowsy state to the annotation framework. A description for this state could, for example, be the one used by Anders and Chalemian (1974), that is, “relative immobility, absence of focused attention, and opening and closing of the eyelids.” A new study and/or re‐evaluation of the current data would be needed to re‐validate the updated framework.

The pilot study to validate our framework has a number of strengths and weaknesses. First, an important drawback is the number of participants. Although we based the framework itself on an acceptable number of observations, for the validation study data from only 10 infants could be included. Follow up work with a large population (e.g., >100 infants) is necessary to substantiate the findings reported here. Second, the data the framework was based on were collected at night in a home setting, and data for the pilot study were collected during the day in a laboratory setting. This difference in time of day and setting may translate into differences in infant wake/sleep patterns. However, except for sleep duration we did not observe any such differences, for example, amount of time in each state was similar, transitions were similar, etc. Thus, our framework is suitable for both home and lab settings. A strong point of the pilot study is that we were able to make a comparison between our framework and the golden standard, PSG, in a controlled, laboratory environment.

As mentioned in the methods section comparing AS and QS as defined in BSA to REM and NREM as defined in PSG was not straightforward. That is, AS is defined as a sleep period during which an infant's eyes are closed, there are eye and body movements, and respiration is irregular. QS is defined as a sleep period during which an infant's eyes are closed, there is lack of eye and body movements, and respiration is regular. Following these definitions, QS cannot be directly compared to NREM sleep, since for the N1 and N2 types of NREM sleep movements and irregular respiration are common (Kirjavainen et al., 1996; Shimohira et al., 1998; Wilde‐Frenz & Schulz, 1983), and respiration is often irregular (Kirjavainen et al., 1996). That a direct comparison is difficult is also visible in our results, where the kappa scores for comparing BSA to PSG according to Wake|REM^+^|NREM^−^ were much higher than those for Wake|REM|NREM. Thus, QS is closer to NREM^−^ than to NREM. For using QS to annotate NREM, one could allow for some movement during quiet sleep (e.g., “an occasional startle”), which is what authors such as Prechtl (1974) and Anders and Chalemian (1974) have done. Then, however, the question arises how to quantify “some” movements, and when do these movements represent quiet/NREM sleep, and when active/REM sleep? Based on the literature and on our experiences we are unsure whether it is possible to reliably compare AS and QS to REM and NREM (N1 + N2 + N3), respectively, without the advantage of EEG. Since this question falls outside the scope of our study, we suggest dedicating future research efforts to quantifying and formalizing the type and quality of movements made during REM/AS and NREM/QS, for which observations from the current framework could form the basis.

Although the unobtrusive nature of the BSA framework makes it suitable for observing sleep in preterm infants, an important question is whether it can be used for the classification of sleep states in this population. For example, preterm infants spend more time in REM‐sleep than term‐born infants (Grigg‐Damberger et al., 2007). Also, sleep in preterm infants is characterized by episodes of indeterminate sleep (Lehtonen & Martin, 2004; Parmelee et al., 1967) which is not defined as a sleep state in the BSA framework. It may be possible to annotate intermediate episodes as AS, but research is necessary to verify whether this is a valid classification. In the same vein, our annotation framework has not yet been validated for infants with other health problems, sleep‐related disorders, or developmental problems. Since these affect sleep (see e.g., Krakowiak et al., 2008; Wiggs & France, 2000) adjustments to the BSA framework may be necessary to be able to reliably classify sleep in infants with these problems. Future work could focus on the type and quality of movements and respiratory patterns per clinical population, and chart potential differences in comparison with healthy populations.

Another remaining question concerns V. According to Prechtl (1974) the main characteristic of this state is vocalization or crying. Although he describes what the vector for V looks like, he does not explicitly specify whether vocalization occurs during periods of sleep, during wakefulness, or during both. Other authors classify vocalization as a waking state (Anders, 1978; Anders & Chalemian, 1974; Anders et al., 1971; Thoman & Tynan, 1979). However, we do not think this is a correct classification. During our annotation work we encountered nocturnal episodes in which an infant was moaning or crying, but with their eyes closed and their movements corresponding to those characteristic of AS. This made us conclude that vocalizations can also happen during sleep. Thus, if desired, an annotator can choose to annotate V not just as a separate state, but in addition to one of the other states. Note that since in the lab study vocalization only occurred during wakefulness, for the current analyses we followed Anders et al. (1971), and treated vocalization as an awake state.

As mentioned earlier in this paper, while using Prechtl's method for annotating our data, we noticed that infants sometimes moved differently than described by Prechtl. An interesting observation here is that we did not observe startles in QS. A possible explanation is the age of our population. That is, Prechtl developed his method for sleep annotations in newborns—that is, a child aged 1 to 28 days—and the infants in our study were 173 days on average. This explanation is in line with findings from Curzi‐Dascalova and Plassart (1978), who studied a cross‐sectional infant cohort, and for QS reported a decrease in startles with increasing age. Also, in a longitudinal study with both premature and term infants Hakamada et al. (1981) found that the number of startles during QS decreased over time. Thus, although for newborns startles during QS appear to be common, for older infants they may no longer make part of their behavioral repertoire during sleep. Future cross‐sectional or longitudinal work could shed more light on this, and could also allow a broader analysis on developmental changes in sleep state parameters.

In relation to that, an additional direction for future research is to study how the behavioral patterns we have described here link to EEG, and to transitions. For example, a larger study could investigate which brain mechanisms and patters underlie the specific behaviors for each state. To enrich such analyses, an exploration of patterns underlying and relating to transitions between states could be included. That is, while following guidelines from the Pediatric Task Force we did not include them here, transitions may have a role in sleep development that justifies their closer inspection. An approach comprising the above can help increase understanding of how behavioral patterns, sleep states, and physiology relate to each other and change over development.

In summary, the current paper presents a behavior‐based framework, which can be used for classifying sleep data unobtrusively collected from healthy infants in their first year of life. The key to using this framework is combining data from different modalities, taking the context into account, and paying close attention to infant movements and respiration during sleep. Data from a pilot study showed that the framework yields results comparable to those acquired by annotation according to PSG, the gold standard for annotation of sleep in infants. Future work with a larger cohort is necessary for further validating this framework, and with clinical populations for determining whether it can be generalized to these populations as well.

## CONFLICT OF INTEREST

The authors declare no conflict of interest. At the time the studies were conducted, and at the time of writing the authors were employed by Philips Electronics Nederland BV.

## AUTHOR CONTRIBUTION

R.O contributed to conceptualization, methodology, study execution, annotation, framework development, and writing—original draft preparation & revision. X.L. contributed to methodology, data analysis, visualization, writing—review and editing. J.W. contributed to framework development and writing—review and editing.
